# Transcription factor target prediction using multiple short expression time series from Arabidopsis thaliana

**DOI:** 10.1186/1471-2105-8-454

**Published:** 2007-11-18

**Authors:** Henning Redestig, Daniel Weicht, Joachim Selbig, Matthew A Hannah

**Affiliations:** 1 Max Planck Institute for Molecular Plant Physiology, Am Mühlenberg 1, D-14476 Potsdam-Golm, Germany; 2 University of Potsdam, Am Neuen Palais, D-14469, Potsdam, Germany

## Abstract

**Background:**

The central role of transcription factors (TFs) in higher eukaryotes has led to much interest in deciphering transcriptional regulatory interactions. Even in the best case, experimental identification of TF target genes is error prone, and has been shown to be improved by considering additional forms of evidence such as expression data. Previous expression based methods have not explicitly tried to associate TFs with their targets and therefore largely ignored the treatment specific and time dependent nature of transcription regulation.

**Results:**

In this study we introduce CERMT, Covariance based Extraction of Regulatory targets using Multiple Time series. Using simulated and real data we show that using multiple expression time series, selecting treatments in which the TF responds, allowing time shifts between TFs and their targets and using covariance to identify highly responding genes appear to be a good strategy. We applied our method to published TF – target gene relationships determined using expression profiling on TF mutants and show that in most cases we obtain significant target gene enrichment and in half of the cases this is sufficient to deliver a usable list of high-confidence target genes.

**Conclusion:**

CERMT could be immediately useful in refining possible target genes of candidate TFs using publicly available data, particularly for organisms lacking comprehensive TF binding data. In the future, we believe its incorporation with other forms of evidence may improve integrative genome-wide predictions of transcriptional networks.

## Background

Transcriptional regulation is essential for all eukaryotes and is central to the complex development and environmental responses of higher organisms. The identification of transcription factors (TFs), TF-target genes and transcriptional regulatory networks is therefore of fundamental importance for biology. The ability of TFs to modify the expression of many physiologically important target genes has made them attractive targets for biotechnology [1,2]. Traditionally, experimental approaches have sought to identify TF-targets by measuring gene expression in loss- or gain-of-function mutants, whilst TF binding to their target promoters has been measured using gel-shift assays, co-transfection assays or chromatin-immunoprecipitation (ChIP). With the arrival of genome-scale technologies, approaches have been scaled up to allow for the unbiased identification of either genes with altered expression in TF mutants using expression profiling, or promoters and other genomic sequences that are bound by a TF *in vivo *by hybridizing ChIP samples to DNA microarrays (ChIP-chip) [3,4].

However, phenotypes of TF mutants are the product of the combination of temporal, developmental and genetic interactions with the altered gene function. Target identification may therefore be confounded by factors such as redundancy, pleiotropic overlap, severe developmental phenotypes or lethality (e.g. [5,6]). The use of inducible expression or inducible nuclear targeting of the TF may overcome these limitations but such systems have been rarely used and can also lead to secondary effects [7,8]. Likewise, genome-wide location data for TF binding from ChIP-chip experiments does not provide definitive evidence of target regulation. Observed DNA binding is not always sufficient to accurately predict a regulatory interaction [4,9] as it may be related to a process other than transcriptional control of gene expression, or simply be biologically irrelevant. In yeast, ChIP-chip has been comprehensively applied to all 203 predicted TFs. However, such data provides only a snapshot of the complete regulatory network as interactions are dependent on many variables such as the cell type, genetic background and developmental stage of the organism, and the timing and type of environmental or biological stimuli [10]. In the case of higher eukaryotes, which have an order of magnitude greater diversity of both TFs [11] and potential targets, mapping the regulatory network would require a currently unfeasible amount of time and resources. The central role of TFs and the limitations of the available data have together generated considerable interest in the computational prediction of TF-targets and regulatory networks. Applied simply, genes with altered expression in a TF mutant may be filtered by the presence or absence of a binding motif for the TF or for those showing a similar treatment-response to the TF (e.g. [12]). More complex algorithms have been used to improve the target prediction accuracy by combining ChIP-chip data with other resources such as phylogeny, TF binding motifs, co-expression data, or a combination of these [4,10,13,14]. The power of combining multiple forms of evidence was recently demonstrated by Beyer and coworkers, who, by using eight forms of evidence, were able to predict previously unknown TF-binding interactions that could subsequently be proven by new, condition-directed, ChIP-chip experiments [10]. In the absence of more comprehensive ChIP-chip data, the application of these methods to higher eukaryotes is not yet feasible. One form of evidence that is also widely available for higher eukaryotes is co-expression data, which has become commonly used in computational biology since the increase in public availability of microarray data. Several tools that support such analyses have been developed (e.g. [15-18]). These analyses have been used to identify additional components of enzymatic modules [19] and assign specific functions to generalist enzymes [20]. Co-expression relationships may also support the known regulation of target genes by a TF [20]. However, such examples are limited and the utility of co-expression data to predict targets of a given TF in an unsupervised fashion has not been explored. Given the importance of translocation and post-translational modification (e.g. [21,22]) this is understandable. However, TFs and their targets do tend to be co-expressed and by applying methods that overcome problems such as time shifts [23] and conditional responses [24], gene expression data can be used as a proxy to measure TF activity. Shi et al. [14] recently demonstrated this by using expression data from multiple time series and considering the possibility of time shifts when predicting TF-targets. By modeling the known regulatory relationships, they estimated treatment specific time scales which they then used to estimate the correct time shifts for predicting novel interactions. Their method depends on comprehensive prior knowledge in the form of large scale ChIP-chip data [4], and is therefore not yet applicable to organisms for which such resources are still unavailable. Furthermore, given the demonstrated condition dependency of ChIP-chip data and that its utility for inferring general properties of TF – TF-target interactions is not fully assessed, it is unclear whether the ChIP-chip data now becoming available will be useful beyond the directly studied TFs. Hence, it is of interest to examine the performance of methods that do not require prior information about regulatory interactions.

Lacking appropriate training data, one has to resort to fully unsupervised (clustering) approaches. Several clustering methods that include the possibility of time shifts for gene expression data (e.g. [25-27]) have been described, but all of them take an *ab initio *approach by not using the information of which genes are supposed to be TFs, and are therefore unsuitable for querying the data for targets for a particular TF. Recently, Heard and coworkers also proposed a clustering method that can use multiple gene expression time series [28], but without considering time shifts.

A prerequisite for incorporating temporal information into TF-target gene prediction is the selection of an appropriate dataset and the concomitant selection of a model organism. As will be discussed, the AtGenExpress consortium's stress series dataset for the model plant *Arabidopsis thaliana *was selected as the most technically and biologically appropriate. In particular, TF-target prediction in higher eukaryotes is appealing due to the aforementioned unfeasible task of comprehensive mapping of all TF-target gene interactions, meaning that such a method could have immediate biological application.

We developed a method to identify potential TF-target genes as those responding strongly to the same stimuli as their controlling TF(s) in a coordinate temporal response. Initially, simulated data with predefined TF-target gene expression relationships showed that, by selecting treatments and incorporating temporal information, our algorithm can improve performance as compared to conventional co-expression based methods. We then applied the method to identify known TF – target gene relationships, as experimentally determined, using expression profiling on TF mutants. These data revealed that the method was useful to enrich targets for a diverse set of experimentally determined TF – target gene relationships. Furthermore, for half of the studied TFs, the enrichment of true targets among extracted genes was sufficient to obtain usable numbers of high-confidence target genes. By looking at a large set of annotated TFs, we also observed that the targets predicted using our approach are more enriched with both functional annotations and putative cis-elements compared to those obtained by conventional methods, hence, indicating a higher biological relevance.

We envisage our method could be immediately useful in narrowing the search for target genes of candidate TFs using publicly available data either through direct prediction or by filtering data obtained by expression profiling of TF mutants. This would be particularly applicable for organisms lacking comprehensive TF binding data. We show that considering other evidence has the potential to improve the methods performance and, in the future, we believe its incorporation into methods using multiple forms of evidence may improve integrative genome-wide predictions of transcriptional networks.

## Results and discussion

### Covariance based extraction of regulatory targets using multiple time series

In a simple scenario, assuming full transcriptional control of gene expression, the target genes will have the same characteristic expression pattern as the regulating TF itself, although possibly shifted forward in time. However, other genes can have similar expression pattern as a direct response to an applied treatment even though they are unrelated in a regulatory sense. In order to separate such co-expression from the more interesting co-regulation one has to look at many different time series of the same system but exposed to different perturbations.

A direct approach to utilize such data for co-expression analysis is to concatenate the available time courses and compute correlations based on the constructed pseudo time series as was done in the co-expression databases CSB.DB and ATTED-II [16,18]. However, there are two main conceptual problems with this approach. Biological interaction patterns are very dynamic and genes that are co-regulated in one condition can share little resemblance in the next, particularly if they happened to regulated by more than one TF [29]. Thus, the inclusion of an experiment in which the TF is not active could theoretically worsen predictions. The second problem is that transcriptionally controlled regulation is delayed, so if the resolution of the time series is high enough then the TF will only be correlated with its target in a time shifted manner. Moreover, this delay might not be the same across the different treatments because even though the studied *physical *time frame is the same, the *biological *time frame might differ. For example, transcription (like all other chemical reactions) is affected by temperature and so the time shift between the TF and its target is likely to be different under high versus low temperature treatments. Ergo, the problem is two-fold. In order to make good predictions of plausible targets we propose that it is necessary to, from the total set of considered treatments, *I*: pick the 'right' subset of treatments and *II*: introduce the 'right' time shift for these. Finally, the use of the correlation coefficient implies that the scale of changes in gene expression is irrelevant and only the shape matters. To overcome the background noise from the numerous untranscribed genes one usually applies some sort of variance threshold. Here, we instead experiment with using the covariance, which pays attention to *both *shape and magnitude, instead of correlation and thus assume that big changes are more relevant than small changes. To summarize the previous section, the following assumptions will lay the ground for our approach:

• The expression of the true target genes are transcriptionally controlled by the investigated TF.

• The TF-targets have a similar (covariant) response to the TF but may show a treatment dependent delay.

• Genes can be part of more than one regulon so any treatments in which the TF does not respond are also not informative.

• Because not all genes are transcribed at the same time, the sought TF-targets will have higher variance than the bulk of genes.

**Figure 1:**
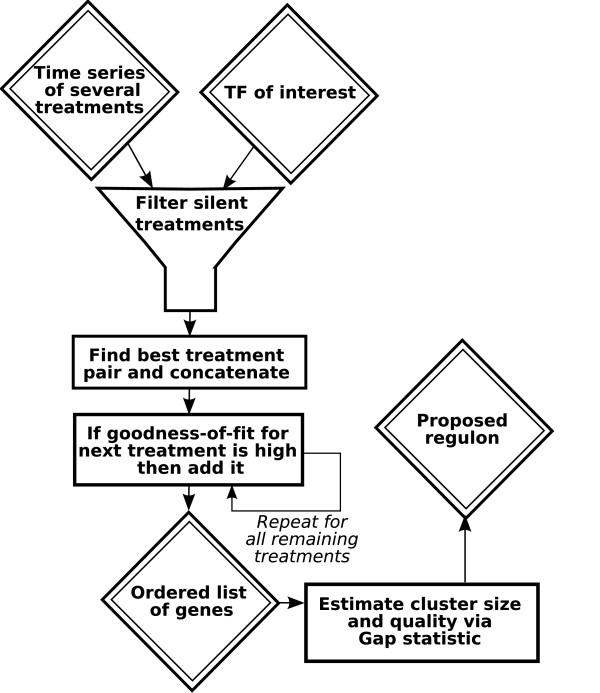
**A flowchart of the CERMT algorithm**. The input is a set of microarray time series for several treatments and a transcription factor (TF) of interest. First the treatments in which the TF does not respond are removed. Then a pair of treatments are selected for which the same genes are highly covariant with the TF. The rest of the treatments are then searched and added or discarded depending on a goodness-of-fit test. Finally a cut-off for the gene list ordered by their covariance with the, possibly time shifted, TF in the selected treatments is estimated via the Gap statistic.

Given a set of gene expression time series and a TF of interest, the output of the proposed method is a cluster of co-expressed genes that, given the assumptions above, look like they are controlled by the TF of interest. Because the cluster is directly associated with a known TF, we will instead refer to it as a predicted *regulon*. Figure 1 shows a flow scheme of the proposed algorithm, and below we outline the main strategies.

#### Method outline

The method we suggest predicts regulons by first removing the time series in which the TF does not respond. We do this by only including treatments in which the TF exceeds two thresholds; the overall maximum response of the TF and the maximum difference between the TF of the stressed plant and the same TF under control conditions. The latter is necessary to account for the extensive diurnal expression changes of *Arabidopsis *[30].

The remaining treatments are organized in a three dimensional expression matrix, *X *= *x*_*i*, *j*, *k*_, with measurements from *n*_*t *_different time points (*i*), *n*_*g *_genes (*j*) and *n*_*p*_different treatments (*k*). Following the stipulated assumptions, we rank the genes according to how strongly associated they are with the TF, by seeking a set of treatments, *m*, and corresponding lags, *l*, for which the covariance

cj,k,lk=∑i=1nt−lk(yi,k−y¯k,lk)(xi+lk,j,k−x¯j,k,lk)nt−lk,
 MathType@MTEF@5@5@+=feaafiart1ev1aaatCvAUfKttLearuWrP9MDH5MBPbIqV92AaeXatLxBI9gBaebbnrfifHhDYfgasaacPC6xNi=xI8qiVKYPFjYdHaVhbbf9v8qqaqFr0xc9vqFj0dXdbba91qpepeI8k8fiI+fsY=rqGqVepae9pg0db9vqaiVgFr0xfr=xfr=xc9adbaqaaeGacaGaaiaabeqaaeqabiWaaaGcbaGaem4yam2aaSbaaSqaaiabdQgaQjabcYcaSiabdUgaRjabcYcaSiabdYgaSnaaBaaameaacqWGRbWAaeqaaaWcbeaakiabg2da9maaqahabaqcfa4aaSaaaeaacqGGOaakcqWG5bqEdaWgaaqaaiabdMgaPjabcYcaSiabdUgaRbqabaGaeyOeI0IafmyEaKNbaebadaWgaaqaaiabdUgaRjabcYcaSiabdYgaSnaaBaaabaGaem4AaSgabeaaaeqaaiabcMcaPiabcIcaOiabdIha4naaBaaabaGaemyAaKMaey4kaSIaemiBaW2aaSbaaeaacqWGRbWAaeqaaiabcYcaSiabdQgaQjabcYcaSiabdUgaRbqabaGaeyOeI0IafmiEaGNbaebadaWgaaqaaiabdQgaQjabcYcaSiabdUgaRjabcYcaSiabdYgaSnaaBaaabaGaem4AaSgabeaaaeqaaiabcMcaPaqaaiabd6gaUnaaBaaabaGaemiDaqhabeaacqGHsislcqWGSbaBdaWgaaqaaiabdUgaRbqabaaaaaWcbaGaemyAaKMaeyypa0JaeGymaedabaGaemOBa42aaSbaaWqaaiabdsha0bqabaWccqGHsislcqWGSbaBdaWgaaadbaGaem4AaSgabeaaa0GaeyyeIuoakiabcYcaSaaa@7073@

is maximized for the genes in the sought regulon in all *k *∈ *m*. In (1), x¯j,k,lk
 MathType@MTEF@5@5@+=feaafiart1ev1aaatCvAUfKttLearuWrP9MDH5MBPbIqV92AaeXatLxBI9gBaebbnrfifHhDYfgasaacPC6xNi=xH8viVGI8Gi=hEeeu0xXdbba9frFj0xb9qqpG0dXdb9aspeI8k8fiI+fsY=rqGqVepae9pg0db9vqaiVgFr0xfr=xfr=xc9adbaqaaeGacaGaaiaabeqaaeqabiWaaaGcbaGafmiEaGNbaebadaWgaaWcbaGaemOAaOMaeiilaWIaem4AaSMaeiilaWIaemiBaW2aaSbaaWqaaiabdUgaRbqabaaaleqaaaaa@3504@ is the average expression of gene *j *in treatment *k *shifted backward by *l*_*k *_time points and y¯k,lk
 MathType@MTEF@5@5@+=feaafiart1ev1aaatCvAUfKttLearuWrP9MDH5MBPbIqV92AaeXatLxBI9gBaebbnrfifHhDYfgasaacPC6xNi=xH8viVGI8Gi=hEeeu0xXdbba9frFj0xb9qqpG0dXdb9aspeI8k8fiI+fsY=rqGqVepae9pg0db9vqaiVgFr0xfr=xfr=xc9adbaqaaeGacaGaaiaabeqaaeqabiWaaaGcbaGafmyEaKNbaebadaWgaaWcbaGaem4AaSMaeiilaWIaemiBaW2aaSbaaWqaaiabdUgaRbqabaaaleqaaaaa@32C9@ is the average expression of the TF in treatment *k *truncated by *l*_*k *_time points.

Finding the optimal solutions for *m *and *l *is difficult as it would require knowledge about the identity of at least some of the true targets. This information is unavailable in our setting and therefore we design the following greedy heuristic. Assuming that the regulon is large enough and under control of the TF in at least two treatments, *m*_1 _and *m*_2_, after time lags *l*_1 _and *l*_2_, then the product of the two corresponding covariance vectors will be high for a good pair of treatments and lags. The product of the covariance vectors from two lagged treatments is defined as:

C(k1,k1,l1,l2)=∑j=1ngcj,k1,l1×cj,k2,l2.
 MathType@MTEF@5@5@+=feaafiart1ev1aaatCvAUfKttLearuWrP9MDH5MBPbIqV92AaeXatLxBI9gBaebbnrfifHhDYfgasaacPC6xNi=xI8qiVKYPFjYdHaVhbbf9v8qqaqFr0xc9vqFj0dXdbba91qpepeI8k8fiI+fsY=rqGqVepae9pg0db9vqaiVgFr0xfr=xfr=xc9adbaqaaeGacaGaaiaabeqaaeqabiWaaaGcbaGaem4qamKaeiikaGIaem4AaS2aaSbaaSqaaiabigdaXaqabaGccqGGSaalcqWGRbWAdaWgaaWcbaGaeGymaedabeaakiabcYcaSiabdYgaSnaaBaaaleaacqaIXaqmaeqaaOGaeiilaWIaemiBaW2aaSbaaSqaaiabikdaYaqabaGccqGGPaqkcqGH9aqpdaaeWbqaaiabdogaJnaaBaaaleaacqWGQbGAcqGGSaalcqWGRbWAdaWgaaadbaGaeGymaedabeaaliabcYcaSiabdYgaSnaaBaaameaacqaIXaqmaeqaaaWcbeaakiabgEna0kabdogaJnaaBaaaleaacqWGQbGAcqGGSaalcqWGRbWAdaWgaaadbaGaeGOmaidabeaaliabcYcaSiabdYgaSnaaBaaameaacqaIYaGmaeqaaaWcbeaaaeaacqWGQbGAcqGH9aqpcqaIXaqmaeaacqWGUbGBdaWgaaadbaGaem4zaCgabeaaa0GaeyyeIuoakiabc6caUaaa@5B78@

**Figure 2:**
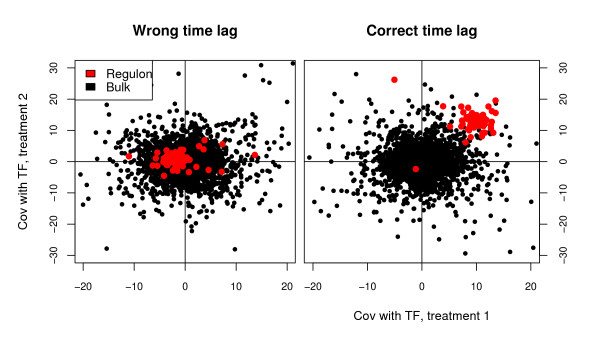
**The covariance between a simulated transcription factor (TF) and all other genes in two different treatments**. With no time shift (left panel) the true regulon (red points) has low covariance with the TF in both treatments. When the expression of the TF has been shifted forward (right panel) the correct number of time points it becomes highly covariant with its regulon and (2) increases.

If we only consider a relatively small number of possible lags, we can set the seed pair of treatments to:

{m^1,m^2,l^1,l^2}=arg⁡max⁡m1,m2,l1,l2C(m1,m2,l1,l2)
 MathType@MTEF@5@5@+=feaafiart1ev1aaatCvAUfKttLearuWrP9MDH5MBPbIqV92AaeXatLxBI9gBaebbnrfifHhDYfgasaacPC6xNi=xI8qiVKYPFjYdHaVhbbf9v8qqaqFr0xc9vqFj0dXdbba91qpepeI8k8fiI+fsY=rqGqVepae9pg0db9vqaiVgFr0xfr=xfr=xc9adbaqaaeGacaGaaiaabeqaaeqabiWaaaGcbaGaei4EaSNafmyBa0MbaKaadaWgaaWcbaGaeGymaedabeaakiabcYcaSiqbd2gaTzaajaWaaSbaaSqaaiabikdaYaqabaGccqGGSaalcuWGSbaBgaqcamaaBaaaleaacqaIXaqmaeqaaOGaeiilaWIafmiBaWMbaKaadaWgaaWcbaGaeGOmaidabeaakiabc2ha9jabg2da9iGbcggaHjabckhaYjabcEgaNnaaxababaGagiyBa0MaeiyyaeMaeiiEaGhaleaacqWGTbqBdaWgaaadbaGaeGymaedabeaaliabcYcaSiabd2gaTnaaBaaameaacqaIYaGmaeqaaSGaeiilaWIaemiBaW2aaSbaaWqaaiabigdaXaqabaWccqGGSaalcqWGSbaBdaWgaaadbaGaeGOmaidabeaaaSqabaGccqWGdbWqcqGGOaakcqWGTbqBdaWgaaWcbaGaeGymaedabeaakiabcYcaSiabd2gaTnaaBaaaleaacqaIYaGmaeqaaOGaeiilaWIaemiBaW2aaSbaaSqaaiabigdaXaqabaGccqGGSaalcqWGSbaBdaWgaaWcbaGaeGOmaidabeaakiabcMcaPaaa@61F3@

by exhaustively trying all pairs of treatments and lags. Figure 2 exemplifies the idea. The sought regulon co-varies with its TF in two treatments at a certain time shift and causes (2) to reach its maximum for these.

Once the best pair has been found we create a summary of the two treatments by concatenating the measurement vectors to obtain a pseudo treatment with 2*n*_*t *_- *l*_1 _- *l*_2 _time points and again calculating the covariance between the TF and the rest of the genes according (1).

By ordering the genes after their covariance with the TF we assume that the proposed regulator is an inducer. If desired (see Section 'Target accuracy and input specificity'), repressed targets can be extracting by reversing the search order, i.e. by replacing cj,k,lk
 MathType@MTEF@5@5@+=feaafiart1ev1aaatCvAUfKttLearuWrP9MDH5MBPbIqV92AaeXatLxBI9gBaebbnrfifHhDYfgasaacPC6xNi=xH8viVGI8Gi=hEeeu0xXdbba9frFj0xb9qqpG0dXdb9aspeI8k8fiI+fsY=rqGqVepae9pg0db9vqaiVgFr0xfr=xfr=xc9adbaqaaeGacaGaaiaabeqaaeqabiWaaaGcbaGaem4yam2aaSbaaSqaaiabdQgaQjabcYcaSiabdUgaRjabcYcaSiabdYgaSnaaBaaameaacqWGRbWAaeqaaaWcbeaaaaa@34C2@ with -cj,k,lk
 MathType@MTEF@5@5@+=feaafiart1ev1aaatCvAUfKttLearuWrP9MDH5MBPbIqV92AaeXatLxBI9gBaebbnrfifHhDYfgasaacPC6xNi=xH8viVGI8Gi=hEeeu0xXdbba9frFj0xb9qqpG0dXdb9aspeI8k8fiI+fsY=rqGqVepae9pg0db9vqaiVgFr0xfr=xfr=xc9adbaqaaeGacaGaaiaabeqaaeqabiWaaaGcbaGaem4yam2aaSbaaSqaaiabdQgaQjabcYcaSiabdUgaRjabcYcaSiabdYgaSnaaBaaameaacqWGRbWAaeqaaaWcbeaaaaa@34C2@. This does not affect the initial search for a good pair of treatments and lags.

In order to investigate if there are more treatments for which (1) is high for the same genes, we order the remaining treatments according to (2) by setting *m*_1 _to the artificial pseudo treatment. If a cross-validation based goodness-of-fit test suggests that the next treatment is useful, it is added and the procedure is reiterated.

By selecting treatments and lags we construct a pseudo-time series in which the top-ranked genes have high covariance with the TF. This would be true even if all of the expression data were completely independent of the TF. Therefore, we must investigate how likely it is to observe regulons of the same quality from randomized data. We did this by adapting the Gap statistic described by Hastie et al. [31] to our problem. The Gap statistic is beneficial as it both provides an estimate of the statistical quality of the proposed regulon, and simultaneously recommends the best number of genes to extract.

### Comparisons with other approaches

Conceptually, our approach differs from other co-expression based methods in that it aims to directly associate a known TF with target genes. As it does not require extensive prior knowledge (i.e. ChIP-chip data), it also differs from a recently described method [14] to identify target genes for known TFs. Methodologically, our method is characterized by two main aspects. Firstly, it incorporates the *a priori *assumption that true TF-targets have higher variance then the bulk of the represented genes by using the covariance instead of correlation. Secondly, it performs a selection of treatments and time shifts to increase overlap between the TF and putative targets. In order to investigate the importance of both of these components we assessed the performance of both the full CERMT approach and a reduced version, CERMT-0, which always uses all treatments without considering any time shifts.

By choosing a different initial pair of treatments, by for example excluding treatments that are previously suspected to be irrelevant, it is possible to obtain different regulons. For simplicity, we will in this study restrict ourselves to consider only one regulon for each TF.

The standard work-horse for detecting co-expression is the Pearson correlation and we therefore compare our results to just concatenating all time series and ranking genes against their correlation with the TF. Here, we refer to this approach as 'Cor'. However, considering the timescale, limited number of samples and relatively controlled conditions, the AtGenExpress dataset may not allow for the best comparison with a correlation based method. We therefore chose ACT [15] to provide a more stringent comparison, which like other co-expression tools [16-18], uses a highly diverse dataset from hundreds of steady state conditions. Repressed targets were sought by ordering the genes after their negative correlation.

To measure performance, we first looked at the threshold-free AUC statistic (area under the ROC curve), but this performed poorly with such grossly different sample sizes (false targets vs. true targets). Therefore, we simply counted the number of true targets among of the top 100 predicted genes, thereby also allowing us to assess the ability of the method to return true targets in the very top of the gene list; an important aspect as experimental validation rapidly becomes infeasible with the number of predicted genes.

### Simulated data

**Figure 3:**
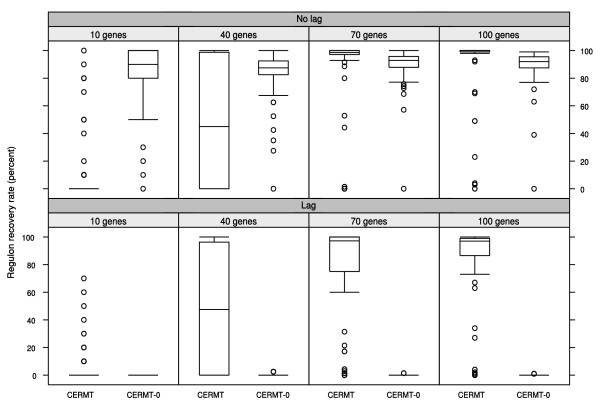
**Boxplot of method performance using simulated data**. Performance was measured as the percentage of the recovered genes (100 × True positives/*n*) in the top 2*n *predicted genes where *n *equals the size of the planted regulon. The simplistic method CERMT-0 does not consider any time lag, makes no selection of treatments and is therefore robust against the size of the planted regulon but for the same reason also fails if there exists a time lag between the TF and its targets. CERMT on the other hand performs poorly if the planted regulon is small, but it is robust against the presence of time lags.

We compared CERMT with its reduced version, CERMT-0, on 100 simulated data sets that contained 10000 genes, six different treatments and seven time points. In three of the treatments, a regulon of varying size was added that followed the pattern of the TF directly or lagged by either 1 or 2 time points. Figure 3 shows boxplots on the percentage of the true positives that were found in the top 2*n *genes, where *n *equals the size of the planted regulon. The performance of CERMT-0 is high if there is no time lag, but, not surprisingly, very poor if we plant a delay. The full CERMT approach performs poorly if the sought regulon is too small as the 'right' treatment pair becomes increasingly diffcult to find with decreasing regulon size. On the other hand, for sufficiently large regulons, CERMT shows good performance regardless of whether response is delayed or not. The size of the smallest detectable regulon decreases with the amount of time points in the experiment (data not shown). Note that though the limit for the minimum regulon size in the simulation seems to be around 50 genes, this estimate is strongly data specific and is not transferable to performance on real data.

### Comparison with over-expression and knock-out experiments using the AtGenExpress dataset

The abiotic stress series from the AtGenExpress project [32] was selected for evaluation of the proposed method. This large data set uses the standardized Affymetrix platform and was all generated in parallel through the coordinated work of five different research groups. It consists of data from root and shoot of 16 days old *Arabidopsis *seedlings exposed to nine different abiotic stresses as well as control conditions, giving a total of 18 different usable time series (loosely referred to as 'treatments'). The seven time points commonly measured in all treatments were selected from each experiment to arrive at an expression matrix with 140 samples. These time points were 0, 0.5, 1, 3, 6, 12 and 24 hours, which approximates a log timescale. For this data, log scaled sampling seems preferable as we noted that a log-linear model yields higher absolute *t*-values than a linear model, thus, a majority of the genes have a more linear response in log-time than in linear time. Non-linear sampling could otherwise have detrimental effects on any time shifting attempts. Considering the response of plant gene expression to various perturbations (e.g. [33,34]), it seems reasonable to assume that this timescale will reveal at least some of the biologically relevant TF-TF-target relationships.

CERMT depends on having expression estimates at the same time points in all treatments. Datasets which do not fulfill this can also be used if common time points are first interpolated, which preferably can be done using sophisticated interpolation strategies such as that proposed by Bar-Joseph et al. [14,35]. Relying solely on gene expression data, we can only expect to extract TF-targets for TFs that actually respond to the applied treatments. Therefore, we conducted a literature study specifically to find experiments investigating the targets of abiotic stress related TFs either by over-expression, knock-out or ChIP-chip experiments, see Additional file 1. Also, in the cases where we could find known motifs for TF's [36-38] we extracted all genes with those motifs in their 500 base upstream regions regarding that gene list as a set of 'targets' to predict. We expect the experimentally obtained lists of TF – target relationships to likely contain many erroneous findings. For example, as many TFs regulate other TFs, it has been pointed out that the effects of constitutive TF mutants, as defined by expression profiling, will likely include those of the regulated TFs [3]. Despite this, we feel this TF target practice is a useful exercise. Firstly, only some of the experimentally predicted targets need to be *true *targets in order for these lists to be useful for the comparison of methods. Secondly, the targets predicted by the method could help in reducing the number of false-positives in experimental predictions. Thirdly, even if all genes represent indirect effects of regulated downstream TFs, these effects may relate to the overall biological function of the candidate TF and so their prediction could still be useful. Previously, a simplified version of the second of these arguments was used to restrict gene lists obtained by over-expressing cold-responsive TFs by also requiring the genes to be cold-responsive [12]. To maximize the number of TF datasets against which we could assess our method, we somewhat loosened our selection from considering only fully transcriptionally regulated individual TFs. We included MBF1c, a transcriptional co-activator [39]; the functionally paired MYC2/MYB2 TFs [40] and HSFA1a/HSFA1b [41]; the functionally redundant CBF1-3 TFs [42] and the post-translationally regulated TFs DREB2A [43] and AREB1 [44]. All TFs considered as pairs/functionally redundant showed very similar expression patterns (data not shown). The inclusion of DREB2A and AREB1 was motivated by their demonstrated parallel transcriptional and post-translational stress regulation [43,44], which should allow their transcription to act as a proxy for their direct target regulation. Their inclusion could therefore validate wider use of the proposed method beyond strictly transcriptionally controlled TFs. Therefore, we do not expect these lists to wholly represent direct TF-target genes, but considering these down-stream effects, there is still utility in their prediction.

#### Target accuracy and input specificity

An important property of a prediction method is that it shows specificity towards the input TF, i.e. the real TF should really be a better input for enriching its own targets than a random TF. To measure this, we ran the algorithm on all 1484 genes in our data set annotated to bin 27.3, 'RNA.regulation of transcription', by the MapMan project and computed an empirical specificity *P*-value, *P*_*spec*_, as the number of random genes that gave the same or better enrichments than the 'true' TF divided by the total number of tested TFs. Known TF families comprise approximately 90% of this annotation bin with the remaining 10% being putative TFs or other regulatory proteins. As our method is not specific to TFs but can also apply to other genes that regulate transcription (e.g. MBF1c) this bin is useful for our purposes, however, in the worst case, *P*_*spec *_will have negligible 10% error. Note that to calculate *P*_*spec *_one has to know the true regulon and it is therefore only applicable for validation purposes.

The upper part of Table 1 shows the performance of the proposed method along with that of the simplistic methods and the conventional co-expression database ACT. In general, it seems that co-expression methods can be used to identify experimental targets of a given TF. The covariance based methods CERMT-0 and CERMT perform better than the others in most cases and CERMT has the overall best performance, although, with the exception of the CBF regulon, the performance increase compared to CERMT-0 is admittedly modest. In half of the cases, the ratio of true targets is sufficient (12–59%) to deliver a usable number of high-confidence target genes. ACT is the only method that ever substantially outperforms the proposed method. Interestingly, it does so for PAP1, for which the other methods perform poorly. This indicates that these targets are better found in a larger, mostly steady state correlation dataset and that the proposed method can be complementary to existing methods. *P*_*spec *_follows the over-representation significance as expected, and are generally not different between the covariance or correlation based methods. Despite this, covariance appears to be inclined towards finding genes that are really regulated rather than noise genes that happen to have the same expression trajectory. Presumably, the benefit of using covariance over correlation will decrease rapidly with increasing number of studied time points. For MBF1c, ZAT12 and CBF, the original studies also reported target genes that were suspected to be repressed by the corresponding TF. The lower part of Table 1 shows the predictions for these target pools, assuming that the TF is a repressor. CERMT finds a significant number of repressed targets in the top 100 extracted genes for CBF and ZAT12. Significant overlap with the repressed targets of MBF1c were only found in the top 200 predicted targets (4 hits, *P *= 0.006). However, as the Gap-statistic and the corresponding recommended cluster size indicates, the statistical significance of the extracted regulons is not convincing. Furthermore, the *P*_*spec *_indicates that the found targets are unspecific to their corresponding TF. It is difficult to assess the utility of the method for extracting repressed genes with only three examples, hence, CERMT should be used with caution when searching for repressed targets. Repression of transcription is only visible using microarrays after subsequent degradation of available mRNA and the observed response could be less coordinate among a regulon than during induction as degradation rate varies between different mRNAs. The time scale is also likely to be quite different compared to that of induced responses. Thus, searching for repression using expression data could very well be more difficult than searching for induced targets.

**Table 1:** Performance on real data

**TF**	**Targetpool**			**Cor**		**ACT**		**CERMT-0**		**CERMT**		**CERMT Diagnostics**		
	Source	*E*	Size	Hits^100^	*P*_*Spec*_	Hits^100^	*P*_*Spec*_	Hits^100^	*P*_*Spec*_	Hits^100^	*P*_*Spec*_	Size	Hits^Gap^	*Gap*
*Induced targets*														

AREB	OX	0.16	28	2	0.03	*1*	0.15	**9**	0.03	**9**	0.04	128	10	0.20
CBF	CRE	14.32	2508	*18*	0.17	*0*	1.00	34	0.10	**58**	0.01	86	53	0.27
	OX	0.82	143	5	0.08	*0*	1.00	19	0.10	**56**	0.00		52	
DREB2A	OX	0.12	21	3	0.03	*1*	0.07	10	0.06	**11**	0.05	244	15	0.04
HSFA2	OX	0.24	42	12	0.00	*0*	1.00	**22**	0.00	20	0.02	76	19	0.09
HY5	ChIP/KO	0.76	133	*0*	1.00	9	0.03	9	0.11	**12**	0.04	902	32	-0.19
	KO	0.69	120	13	0.00	**19**	0.00	14	0.00	14	0.00		46	
	CRE	12.07	2113	22	0.11	**24**	0.05	19	0.31	19	0.30		153	
MBF1c	OX	0.86	150	*0*	1.00	*2*	0.17	*2*	0.36	**4**	0.22	102	4	0.09
MYB2/MYC2	CRE	6.75	1182	*2*	0.46	***6***	0.64	*5*	0.39	*4*	0.42	536	*31*	0.23
	CRE	19.90	3485	31	0.02	*23*	0.32	30	0.07	**32**	0.05		*121*	
	OX	0.15	26	*0*	1.00	*0*	1.00	***1***	0.26	***1***	0.30		*2*	
NAC019	OX	0.08	14	*0*	1.00	*0*	1.00	*0*	1.00	***1***	0.13	33	*0*	0.01
NAC055	OX	0.05	9	*0*	1.00	**2**	0.00	*0*	1.00	*0*	1.00	121	*0*	0.09
NAC072	OX	0.13	23	*0*	1.00	*0*	1.00	2	0.09	**5**	0.04	96	5	0.25
PAP1	OX	0.24	42	*1*	0.08	**8**	0.00	*0*	1.00	*0*	1.00	27	*0*	0.28
ZAT12	OX	0.79	139	3	0.08	*2*	0.17	8	0.19	**10**	0.06	536	19	0.08

*Repressed targets*														

CBF	OX	0.24	43	*0*	1.00	*0*	1.00	*0*	1.00	**2**	0.17	128	2	0.09
MBF1c	OX	0.46	80	*0*	1.00	*0*	1.00	*0*	1.00	*1*	0.47	1000	10	-0.19
ZAT12	OX	0.90	158	*1*	0.18	3	0.03	7	0.16	**11**	0.12	1000	33	0.01

Hit ratios based on real data for the top 100 genes associated with 12 different transcription factors (TFs). Bold entries are the highest values for that target pool, italic entries are insignificant over-representations according to Fisher's exact test (*p *≥ 0.05). Targetpool size is the number of the true targets in our expression set, *E *is the expected number of hits when picking 100 genes genes at random and *P*_*spec *_is an empirical *P*-value indicating the probability that a random TF would give the same or better hit ratio. For CERMT, the best cluster size as indicated by the Gap statistic is shown along with the number of true targets for that regulon size. A large Gap statistic (greater than zero) indicates that the suggested regulon is significantly more related to the expression of the TF than could be expected from the expression of a shuffled TF. Target pools were defined from over-expression experiments (OX), knock-out experiments (KO), ChIP-chip experiments (ChIP) or by all genes carrying a known cis-regulatory element (CRE).

#### Statistical properties of the predicted regulons

**Figure 4:**
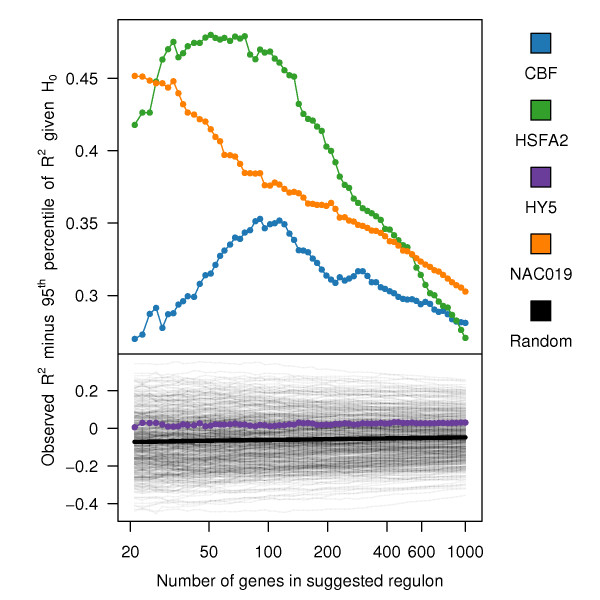
**The Gap curves for four of the examined transcription factors (TFs)**. Shown is the distance between the observed *R*^2^(goodness) of the predicted regulon and the 95^th ^percentile of the null-distribution (which is not shown here). A positive *R*^2 ^means that the regulon is significant on the 5% significance level and the maximum of the Gap curve indicate the best number of genes to include in the regulon. The Gap curves for CBF, NAC072 and AREB are plotted along with the the curves obtained for two-hundred shuffled TFs (thin lines). The shuffled TFs get mostly negative Gap statistics as they lie close the expectation value of the null-distribution. CBF, NAC072 and AREB show very significant Gap curves, the HY5 regulon on the other hand does not.

Figure 4 shows an example plot of the statistical quality of the predicted regulons for four of the examined TFs. The CBF, AREB and NAC072 regulons show convex Gap curves where the maximum indicates the best number of genes to include in the regulon. The observed Gap statistics are greater than zero which indicates that obtaining such a good or better regulon is highly unlikely given that the expression of the TF was independent of the rest of the genes. The HY5 regulon on the other hand, exhibits no stronger connection to its regulon than can be expected from a randomized TF, despite the fact that it contains a significant number of true targets. This could be an effect of the low resolution of the AtGenExpress dataset and the sinusoidal expression pattern of HY5 in response to UV-B stress. Such complex patterns depend on more parameters and are consequently harder to approximate with only seven time points.

#### CERMT can propose biologically interpretable target lists

**Figure 5:**
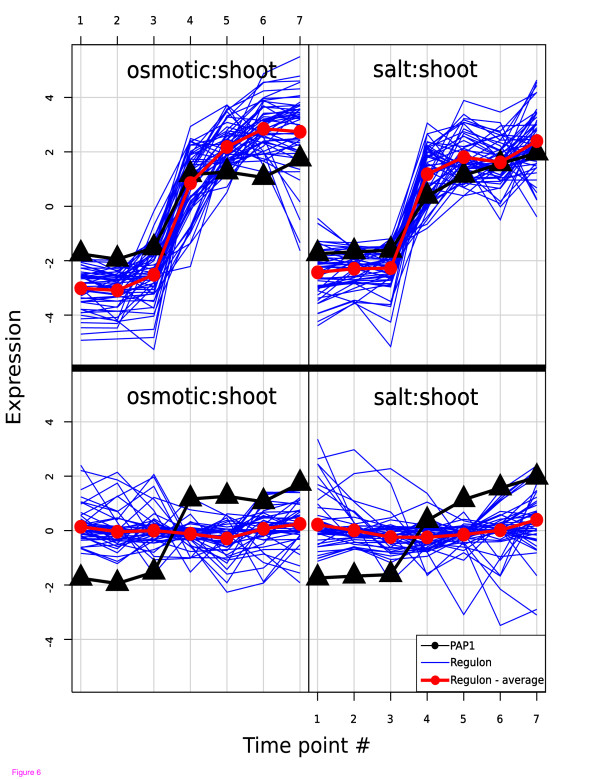
**The PAP regulon**. Comparison of the expression of the CERMT predicted regulon (upper panel) and the over-expression defined regulon [47] (lower panel) versus the expression of the PAP1 transcription factor in the shoot in response to salt and osmotic stress. No time lag was used for the prediction, so there is no overlap between predicted and true regulons. The difference in terms of coherency and variance is pronounced so it is not hard to see why the algorithm is seeded with no time lag instead of the more appropriate lag of two time points. This illustrates an unavoidable problem of TF target prediction based only on gene expression data – there are no unique solutions and the most obvious solution is not necessarily the correct one.

One of the key benefits of the increasing public availability of expression data is the ability to quickly generate hypotheses on gene function. Standard co-expression analyses have yielded several insights that were experimentally validated [19,20]. We therefore investigated the functional insight provided by the CERMT predicted CBF regulon. Remarkably, among the top seven genes there are four COR/LEA genes and one galactinol synthase. The cold-regulated (COR) genes are the defining members of the CBF-regulon as the CBF TFs were first identified through their binding to the C-repeat element present in the promoters of these genes [45]. Galactinol synthase catalyzes the first committed step of raffinose synthesis which is an important component of cold acclimation known to be under the control of the CBF TFs [45]. Overall, the predicted regulon reveals many more known CBF targets including further cold responsive COR genes, enzymes and TFs. These data clearly offer significant biological insight into the central function of the CBF TFs in controlling transcriptional and metabolic changes during cold acclimation. In addition to the predicted target lists, the information about the used treatments shown in Table 2 can also provide useful biological insight into the function of the TF and of the predicted regulon. Several of the studied examples verify known biological information such as CBF's and ZAT12's importance for the response to cold, HY5's for UV, HSFA2's for heat and MBF1c's for heat and osmotic stress [12,39,41,46]. Table 2 also shows which time shifts were used for each TF along with the time shift for which the median covariance between the TF and all the genes in its regulon is maximized in the used treatments. This can be seen as a supervised 'answer' to what the algorithm is trying to predict. It is clear that there often exists a transcriptional time shift for the studied regulons, which justifies one of our primary assumptions. However, the correct time lag is frequently missed by the algorithm. The reason for this becomes apparent when one considers the plots of the over-expression defined regulon for PAP1 and the first 50 genes in the predicted regulon for PAP1, see Figure 5. The difference is glaring so it is not surprising that the true regulon is overlooked. In order to increase performance it would be necessary to use additional resources rather than the gene expression data alone. By, for example, using the information that the deep purple phenotype of the PAP1 over-expresser is due to anthocyanin accumulation and therefore only consider genes involved in flavonoid metabolism [47]. When this information is combined with the *a priori *assumption that there exists a time shift, the algorithm picks out nine of the true targets in its top 100 (Fisher's exact test: *P *= 10^-5^). Including such additional data therefore adds one more TF to those whose hit ratio is sufficient to deliver a usable number of high-confidence target genes. This illustrates an unavoidable problem with gene expression data for TF-target prediction; there are no unique solutions. Given these data sets however, we draw the conclusion that the true regulons often, but far from always, can be discovered with simple statistical functions thus conceptually strengthening the approach by Beyer et al. [10] which integrates many different techniques to boost target predictions.

**Table 2:** The used treatments and time shifts

Regulator	Used treatments	Used shift:Best shift
AREB	osmotic-S, salt-S	0:0 0:2
CBF (Induced)	cold-S, cold-R	2:2 2:2
CBF (Repressed)	cold-S, cold-R, drought-R	2:2 2:2 1:0
DREB2A	genotoxic-R, wounding-S, cold-R, cold-S, osmotic-S	1:0 0:0 2:1 2:1 0:0
HSFA2	drought-R, oxidative-R, oxidative-S, heat-S, heat-R	0:0 0:0 1:0 0:0 0:0
HY5	uvb-R, cold-S, uvb-S	0:0 1:0 0:0
MBF1c (Induced)	osmotic-R, heat-S, heat-R	0:2 0:1 0:0
MBF1c (Repessed)	oxidative-S, cold-R, heat-S, heat-R	1:1 0:0 0:0 0:0
MYC2-MYB2	salt-R, drought-R	0:2 0:1
NAC019	uvb-S, osmotic-R, salt-R osmotic-S, salt-S	0:1 0:0 0:0 0:1 0:0
NAC055	cold-R, osmotic-R, salt-R	0:0 0:1 0:0
NAC072	osmotic-S, salt-S	0:1 0:1
PAP1	osmotic-S, salt-S	0:2 0:2
ZAT12 (Induced)	osmotic-R, cold-R, salt-R	0:2 0:0 0:0
ZAT12 (Repressed)	drought-R, oxidative-R, cold-R, salt-R	0:0 0:0 0:2 0:0

The treatments and time shifts CERMT selected for the real data. Also shown are the 'best' time lags based on maximizing the covariance between the TF and the experimentally determined regulon. These data can be useful for interpreting the biological relevance of the predicted regulon. Treatments ending with 'S' and 'R' are from shoot and root respectively.

#### Biological significance of the predicted regulons for a large set of transcription factors

Having used existing experimentally validated TF-targets to assess the utility of the proposed algorithm for predicting plant regulons, we wished to extend the study to also include less characterized TFs. Although no known targets are available for validating the predictions for such TFs, it is still possible to estimate the statistical significance of the extracted regulons from a biological point of view, and this information can be used to compare the different prediction methods. By searching for significance, we can estimate how randomly chosen a selection of genes seems to be, and, lacking stronger guidelines, we prefer a method which is less random.

A standard method for assessing the biological significance of gene expression clusters is to look at the overlap between the clusters and existing functional annotations [48,49]. The predicted regulons are in a sense also clusters and because targets of a given TF often share biological functions (e.g. [12,47]), we reasoned that the predicted regulons, just like standard gene expression clusters, also should share functional annotations, and that these should be detectable by searching for over-representation.

TFs regulate the expression of their target genes by binding to sequence motifs, i.e. cis-regulatory elements (CREs), in their promoter regions. A group of genes that is more likely to share sequence elements amongst each other is therefore also more likely to be co-regulated. Measuring this likelihood could possibly be done by identifying the best motif and assessing its significance. There are many excellent algorithms for identifying motifs given a set or promoters available (e.g. [50-52]), however, the use of these methods is not feasible for our purpose as most of them are very time consuming and need data specific parameter tuning [53]. Fortunately, we are not directly interested in finding the *correct *motif, but merely to score how reasonable the existence of such a motif is. Therefore we chose a more simplistic method, which was inspired by the enumeration strategy used by the efficacious Weeder algorithm [54], and estimate the likelihood of motif-existence by the number of over-represented nucleotide hexamers. Although it might be naively assumed that a single, highly over-represented motif would be found for each regulon, there are at least two reasons why better regulon prediction is more likely accompanied by an increased number of over-represented motifs. Firstly, the hexamers searched for are redundant or overlapping in sequence, resulting in multiple hits from a single CRE. Secondly, genes may be regulated by more than one CRE, and these 'hitchhiking' elements may also be enriched.

To assess the plausibility of our assumptions about regulon characteristics, we counted the over-represented hexamers and functional annotations in the experimentally defined regulons. Six of the seven target pools that contained more than 30 genes had between 5 to 37 over-represented hexamers and seven had between 3 to 8 over-represented functional annotations, see Additional file 2. In addition, by examining the gene lists annotated to a common known TF binding site by ATCISDB [55], we found that 80% had significantly more over-represented hexamers than could be expected by chance. This strongly indicates that biologically relevant regulons are more likely to contain over-represented annotations and hexamers than random selections of genes. Note that these numbers are dependent on the size of the regulon and are thus not readily transferable to regulons of different sizes.

From the simulated data, we could draw the conclusion that, if the proposed method is better than a simple correlation based measure using concatenated time series, then the improvement would be most apparent when there is a time shift between the TF and its regulon. Therefore, we ran the proposed algorithm, treating the TFs as both inducers and repressors, on each of the 1484 genes in our data set annotated in the MapMan software [56] to bin 27.3, 'RNA.regulation of transcription'. For the 265 and 307 genes that the algorithm chose to introduce a time shift in at least one treatment in induction and repression mode respectively, we counted the over-represented annotations and upstream motifs.

**Figure 6:**
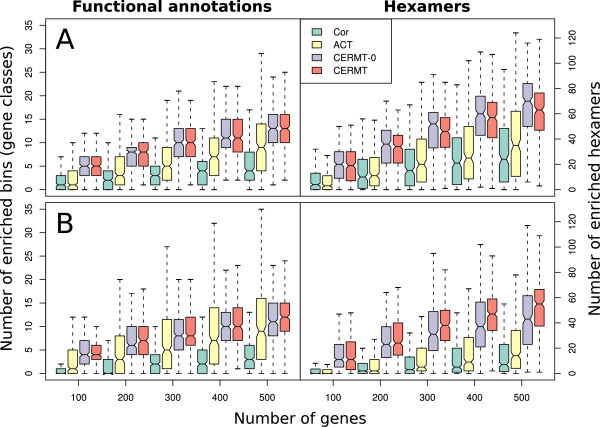
**Characteristics of predicted regulons**. Boxplots of characteristics of predicted regulon. The number of over-represented functional annotations and over-represented hexamers 500 nucleotides upstream in the top 100–500 predicted genes for a selection of transcription factors were counted for each method. The results are shown from both induction mode (A) and (B) repression mode. Truly co-regulated genes share cis-regulatory elements in their promoters and are also likely to share biological function. Due to hexamer redundancy, motif interactions and parallel TF pathways, a higher number of enriched hexamers and functional annotations therefore indicate a higher probability that a group of genes actually is co-regulated. Compared to the previously described methods ACT (Arabidopsis Co-expression tool), Cor (Pearson correlation), the covariance based methods CERMT-0 and CERMT extract genes with more over-represented hexamers and functional annotations.

Figure 6 shows boxplots of the number of significantly over-represented annotations and motifs as a function of the number of extracted genes using the four tested methods. There is strong difference between the correlation and covariance based methods in all four. Obviously, the covariance based methods enrich both more functional annotations and hexamers, and thus extract regulons with more appealing properties than the standard correlation based methods do. No prominent differences could be seen between CERMT and CERMT-0, suggesting that the qualitative differences between these methods was too small to be resolved by this type of enrichment analysis. As indicated by the non-overlapping notches in Figure 6, CERMT-0 enriched slightly more hexamers than CERMT in induction mode, a trend which was reversed and more prominent in repression mode. Having shown that the predicted regulons show significant promoter sequence properties, it is tempting to speculate that a way to further separate overlapping regulons and refine the target lists, could be to integrate CERMT with motif prediction algorithms.

## Conclusion

We designed a method for extracting potential targets to known TFs using gene expression data in the form of multiple time series. The method provides a heuristic for solving the combinatorial problem of selecting informative treatments and appropriate time shifts between the TF and its targets. By maximizing the overlap in covariance between the TF and all other genes in two treatments and then systematically adding further treatments, we not only avoid the need for computationally expensive optimizations, but also increase the interpretability and quality of the predictions.

Using existing experimental data on target associations for twelve TFs, the method showed higher performance than existing steady-state co-expression tools, but indicated that both methods could be complementary. This not only highlighted the utility of the method but also showed that the targets identified by mutant profiling in normal conditions indeed often are highly covariant with the associated TF in a treatment and time dependent fashion in the wild-type plant.

The predicted regulons for unknown TFs also showed appealing properties in terms of enriching both annotations and upstream motifs. These results indicate that the described approach could be used both as a method for exploratory analysis of regulatory relationships of a particular TF, and as a means of obtaining high-confidence subsets from putative target genes identified by mutant profiling or other experimental techniques.

Gene expression based techniques are especially useful for extracting potential targets when no information about the regulatory relationships is available. Such methods can therefore just as well be used for aiding hypothesis generation regarding regulatory properties of e.g. metabolites.

## Methods

### Cross-validation based test for adding further treatments

In order to investigate if there are more treatments for which (1) is high for the same genes, we order the remaining treatments according to (2) by setting the first treatment to the artificial pseudo treatment. For the remaining treatments we measure the goodness-of-fit by estimating the change in predictive performance between the one-component partial least squares (PLS) regression model [57] predicting the expression of the TF from all other genes including the new treatment, *y" *= *X"B *+ *E"*, versus the old one, *y' *= *X'B *+ *E'*, where *B *is the vector with regression coefficients and *E *the residual matrix. The predictive capability is measured by calculating the *Q*^2 ^statistic using repeated five-fold cross-validation. By using Student's *t*-test we test the hypothesis *H*_0 _: *Q*^2' ^> *Q*^2" ^(i.e. including the next treatment led to a decrease in predictive performance) and only include the treatments where we fail to reject. *Q*^2 ^is defined as following:

Q2=1−∑i=1k(y^i−yi)2∑i=1kyi2.
 MathType@MTEF@5@5@+=feaafiart1ev1aaatCvAUfKttLearuWrP9MDH5MBPbIqV92AaeXatLxBI9gBaebbnrfifHhDYfgasaacPC6xNi=xI8qiVKYPFjYdHaVhbbf9v8qqaqFr0xc9vqFj0dXdbba91qpepeI8k8fiI+fsY=rqGqVepae9pg0db9vqaiVgFr0xfr=xfr=xc9adbaqaaeGacaGaaiaabeqaaeqabiWaaaGcbaGaemyuae1aaWbaaSqabeaacqaIYaGmaaGccqGH9aqpcqaIXaqmcqGHsisljuaGdaWcaaqaamaaqadabaGaeiikaGIafmyEaKNbaKaadaWgaaqaaiabdMgaPbqabaGaeyOeI0IaemyEaK3aaSbaaeaacqWGPbqAaeqaaiabcMcaPmaaCaaabeqaaiabikdaYaaaaeaacqWGPbqAcqGH9aqpcqaIXaqmaeaacqWGRbWAaiabggHiLdaabaWaaabmaeaacqWG5bqEdaqhaaqaaiabdMgaPbqaaiabikdaYaaaaeaacqWGPbqAcqGH9aqpcqaIXaqmaeaacqWGRbWAaiabggHiLdaaaiabc6caUaaa@4DA9@

PLS is designed for developing models with strong predictive performance, although this is not our direct interest, it is suitable here as it is desirable to find a set of genes of undefined size that are strongly related to a given TF for *all *treatments used. *Q*^2 ^will not increase when a treatment is added that requires high regression coefficients for genes that are unrelated to the TF in the other treatments and it therefore provides a valid, albeit indirect, tool for deciding whether to leave treatments out or not.

Our method does not allow for more than one time shift per treatment. Therefore, we are only interested in looking for targets responding in the same (induced) or opposite (repressed) direction as the TF. Hence, we modified the PLS algorithm slightly to set all negative or positive coefficients respectively, to zero. According to Höskuldsson [58] this does not affect the central properties of the PLS regression.

### Estimation of the regulon size and significance using the Gap statistic

The Gap statistic has previously been proposed as a method for simultaneously choosing a suitable cluster size and assessing its statistical quality [31]. The method works by calculating a goodness-statistic for several different cluster sizes and choosing that which is farthest away from a pre-defined null-distribution. In our setting, the null-distribution is the goodness-statistics of the regulons we obtain when using a random gene whose expression has been shuffled within each treatment. We define statistical quality of a regulon as the amount of its variance that can be directly related to the TF, and measure this by reversing the previous PLS regression model and calculating *R*^2^.

*X*_*j *∈ 1...*k *_= *yB *+ *E*

R2(k)=∑X^j∈1...k2∑Xj∈1...k2
 MathType@MTEF@5@5@+=feaafiart1ev1aaatCvAUfKttLearuWrP9MDH5MBPbIqV92AaeXatLxBI9gBaebbnrfifHhDYfgasaacPC6xNi=xI8qiVKYPFjYdHaVhbbf9v8qqaqFr0xc9vqFj0dXdbba91qpepeI8k8fiI+fsY=rqGqVepae9pg0db9vqaiVgFr0xfr=xfr=xc9adbaqaaeGacaGaaiaabeqaaeqabiWaaaGcbaGaemOuai1aaWbaaSqabeaacqaIYaGmaaGccqGGOaakcqWGRbWAcqGGPaqkcqGH9aqpjuaGdaWcaaqaamaaqaeabaGafmiwaGLbaKaadaqhaaqaaiabdQgaQjabgIGiolabigdaXiabc6caUiabc6caUiabc6caUiabdUgaRbqaaiabikdaYaaaaeqabeGaeyyeIuoaaeaadaaeabqaaiabdIfaynaaDaaabaGaemOAaOMaeyicI4SaeGymaeJaeiOla4IaeiOla4IaeiOla4Iaem4AaSgabaGaeGOmaidaaaqabeqacqGHris5aaaaaaa@4B86@

We then define the Gap statistic as the observed *R*^2 ^minus the 95^th ^percentile of the null-distribution – *R*^2^*.

Gap(k) = R^2 ^(*k*) - *Q*_0.95_(*R*^2^*(*k*))

The recommended regulon size is given by:

k^=arg⁡max⁡kGap(k).
 MathType@MTEF@5@5@+=feaafiart1ev1aaatCvAUfKttLearuWrP9MDH5MBPbIqV92AaeXatLxBI9gBaebbnrfifHhDYfgasaacPC6xNi=xI8qiVKYPFjYdHaVhbbf9v8qqaqFr0xc9vqFj0dXdbba91qpepeI8k8fiI+fsY=rqGqVepae9pg0db9vqaiVgFr0xfr=xfr=xc9adbaqaaeGacaGaaiaabeqaaeqabiWaaaGcbaGafm4AaSMbaKaacqGH9aqpcyGGHbqycqGGYbGCcqGGNbWzdaWfqaqaaiGbc2gaTjabcggaHjabcIha4bWcbaGaem4AaSgabeaakiabbEeahjabbggaHjabbchaWjabcIcaOiabdUgaRjabcMcaPiabc6caUaaa@4026@

Because we use the 95^th ^percentile, a positive Gap curve can directly be translated to a significant regulon at the 5% confidence level.

### Simulation of gene expression data

The gene expression, *x*, at time point *i *∈ {1, 2,..., 7} for gene *j *∈ {1, 2,..., 10000}, in treatment *k *∈ {1, 2,..., 6} was simulated in a naive way as

xi,j,k={N(0,σxj)+cjyi−lk,k+pj,kyi−lk,kif i>lkN(0,σxj)if i≤lk
 MathType@MTEF@5@5@+=feaafiart1ev1aaatCvAUfKttLearuWrP9MDH5MBPbIqV92AaeXatLxBI9gBaebbnrfifHhDYfgasaacPC6xNi=xI8qiVKYPFjYdHaVhbbf9v8qqaqFr0xc9vqFj0dXdbba91qpepeI8k8fiI+fsY=rqGqVepae9pg0db9vqaiVgFr0xfr=xfr=xc9adbaqaaeGacaGaaiaabeqaaeqabiWaaaGcbaGaemiEaG3aaSbaaSqaaiabdMgaPjabcYcaSiabdQgaQjabcYcaSiabdUgaRbqabaGccqGH9aqpdaGabeqaauaabaqaciaaaeaacqWGobGtcqGGOaakcqaIWaamcqGGSaaliiGacqWFdpWCdaWgaaWcbaGaemiEaG3aaSbaaWqaaiabdQgaQbqabaaaleqaaOGaeiykaKIaey4kaSIaem4yam2aaSbaaSqaaiabdQgaQbqabaGccqWG5bqEdaWgaaWcbaGaemyAaKMaeyOeI0IaemiBaW2aaSbaaWqaaiabdUgaRbqabaWccqGGSaalcqWGRbWAaeqaaOGaey4kaSIaemiCaa3aaSbaaSqaaiabdQgaQjabcYcaSiabdUgaRbqabaGccqWG5bqEdaWgaaWcbaGaemyAaKMaeyOeI0IaemiBaW2aaSbaaWqaaiabdUgaRbqabaWccqGGSaalcqWGRbWAaeqaaaGcbaGaeeyAaKMaeeOzayMaeeiiaaIaemyAaKMaeyOpa4JaemiBaW2aaSbaaSqaaiabdUgaRbqabaaakeaacqWGobGtcqGGOaakcqaIWaamcqGGSaalcqWFdpWCdaWgaaWcbaGaemiEaG3aaSbaaWqaaiabdQgaQbqabaaaleqaaOGaeiykaKcabaGaeeyAaKMaeeOzayMaeeiiaaIaemyAaKMaeyizImQaemiBaW2aaSbaaSqaaiabdUgaRbqabaaaaaGccaGL7baaaaa@77B7@

where

σxj∈Γ(1,0.4)+0.2,yi,k∈N(0,1.5),
 MathType@MTEF@5@5@+=feaafiart1ev1aaatCvAUfKttLearuWrP9MDH5MBPbIqV92AaeXatLxBI9gBaebbnrfifHhDYfgasaacPC6xNi=xI8qiVKYPFjYdHaVhbbf9v8qqaqFr0xc9vqFj0dXdbba91qpepeI8k8fiI+fsY=rqGqVepae9pg0db9vqaiVgFr0xfr=xfr=xc9adbaqaaeGacaGaaiaabeqaaeqabiWaaaGcbaqbaeqabiqaaaqaaGGaciab=n8aZnaaBaaaleaacqWG4baEdaWgaaadbaGaemOAaOgabeaaaSqabaGccqGHiiIZcqqHtoWrcqGGOaakcqaIXaqmcqGGSaalcqaIWaamcqGGUaGlcqaI0aancqGGPaqkcqGHRaWkcqaIWaamcqGGUaGlcqaIYaGmcqGGSaalaeaacqWG5bqEdaWgaaWcbaGaemyAaKMaeiilaWIaem4AaSgabeaakiabgIGiolabd6eaojabcIcaOiabicdaWiabcYcaSiabigdaXiabc6caUiabiwda1iabcMcaPiabcYcaSaaaaaa@4E18@

and *c*_*j *_was one or zero depending on if gene *j *was part of the planted regulon or not. The constant *l*_*k *_defined the planted lag for treatment *k*. The lag was either set to zero or allowed to vary between 1 and 2 time points. The parameters for the distribution of *σ*_*y *_were picked to resemble a real world dataset. Tomake the data more illustrative the term *p*_*j*, *k*_*y*_*i*-*l*, *k *_in (9) was added where *p*_*j*, *k *_was one or zero depending on whether or not the gene *j *belonged to a 'masking' regulon in treatment *k*, a non-intersecting group of genes of the same size as the true regulon. Thus, in order to recover the hidden regulon it is necessary to combine information from different treatments.

Simulating time series data using random normal deviates is naive in the sense that the different time points are independent of each other. For this particular application it is however acceptable as the simplification only becomes detrimental when comparing methods that utilize the time series aspect of the data, as for now CERMT does not do this.

### The AtGenExpress data

The data from abiotic stress series of the AtGenExpress project was downloaded from [59] and normalized using the RMA normalization algorithm [60] as provided by the Bioconductor project [61] for the statistical programming environment R [62]. Probesets matching multiple AGI codes or organellar encoded genes were excluded and where multiple probesets matched the same AGI code the original chip design designations were used and superfluous probesets were dropped in order to obtain a bijective mapping for 20872 probesets. Only probesets that received a present call by the MAS5 algorithm for both replica in at least one time point were kept giving a final expression set of 17513 probesets.

Throughout this study, we only considered the time shifts 0, 0.5 and 1 h, as further time shifts would result in relying on too few time points and unrealistically long transcriptional delays.

The thresholds used for judging whether a TF responded to a treatment or not were set to the standard moderate outliers threshold, *Q*_0.75 _+ 1.5 × *IQR*, i.e. the third quartile plus 1.5 times the inter-quartile range, to the distributions of the maximum responses and maximum deviations from the control, given the probes on the arrays with only insignificant expression signals, as judged by the MAS5 algorithm.

### Motif and Bin enrichment

The enrichment of hexamers in predicted regulons was calculated by first building a dictionary with all possible hexamer, minus those that resembled the TATA-box, and counting their occurrences in the 500 base upstream regions of all considered genes. The obtained global distribution was then compared with that of the predicted regulon. *P*-values for over-representation were calculated using the hypergeometric distribution, FDR corrected [63] and over-representation was noted for FDR < 0.05.

The calculation of annotational enrichments was based on the method proposed by Hannah et al. [64], which uses MapMan ontologies [56,65] combined with Fisher's exact test. Bins (gene classes) were counted as significantly enriched if FDR < 0.05. The MapMan annotations were preferred over alternatives such as mappings to Gene Ontology [66] because of its maturity and plant specific scope.

## Availability and requirements

The R package is contains all methods discussed in this paper and the part of the AtGenExpress data as it was used here. It is not organism specific and makes it possible to apply CERMT to other species after collation of the appropriate gene expression time series. For *Arabidopsis thaliana*, we also provide the method as a web-service which allows the user to select the TF of interest, extract and plot the suggested regulon using a fast but simplified version of the proposed algorithm.

**Project name**: cermt

**Project home page**: 

**Operating systems**: Platform independent

**Programming language**: R package with Java based web-interface

**Licence**: GPL v2

**Any restrictions to use by non-academics**: No

## Authors' contributions

HR designed and implemented the methods and wrote the manuscript. DW implemented the web-service. JS provided essential mentoring and supervision to HR. MH initiated and supervised the project, made the literature study and wrote the manuscript. All authors read and approved the final manuscript.

## Supplementary Material

Additional file 1 Literature study of transcription factors and their targets considered in this study. We focused on TFs that have previously been implicated in stress responses, but as not all respond under the conditions used to generate the AtGenExpress dataset, some were therefore excluded from our test set. Wherever multiple genes were knocked out or over-expressed, or where functional redundancy has been implicated, the average of those genes was used. Click here for file

Additional file 2 The numbers of over-represented hexamers and annotations (MapMan bins) in the experimentally defined regulons. With randomly chosen genes we would not expect any over-representation. A clear majority of the larger regulons have several over-represented hexamers annotations. 'OX' indicates that the targets were found using over-expression, 'KO' using knock-out and 'ChIP' using ChIP-chip experiment. Click here for file

## References

[B1] Kasuga M, Liu Q, Miura S, Yamaguchi-Shinozaki K, Shinozaki K (1999). Improving plant drought, salt, and freezing tolerance by gene transfer of a single stress-inducible transcription factor. Nat Biotechnol.

[B2] Garber K (2006). Intellectual property. Decision on NFkappaB patent could have broad implications for biotech. Science.

[B3] Lee TI, Rinaldi NJ, Robert F, Odom DT, Bar-Joseph Z, Gerber GK, Hannett NM, Harbison CT, Thompson CM, Simon I, Zeitlinger J, Jennings EG, Murray HL, Gordon DB, Ren B, Wyrick JJ, Tagne JB, Volkert TL, Fraenkel E, Gifford DK, Young RA (2002). Transcriptional regulatory networks in Saccharomyces cerevisiae. Science.

[B4] Harbison CT, Gordon DB, Lee TI, Rinaldi NJ, Macisaac KD, Danford TW, Hannett NM, Tagne JB, Reynolds DB, Yoo J, Jennings EG, Zeitlinger J, Pokholok DK, Kellis M, Rolfe PA, Takusagawa KT, Lander ES, Gifford DK, Fraenkel E, Young RA (2004). Transcriptional regulatory code of a eukaryotic genome. Nature.

[B5] Gugasyan R, Voss A, Varigos G, Thomas T, Grumont RJ, Kaur P, Grigoriadis G, Gerondakis S (2004). The transcription factors c-rel and RelA control epidermal development and homeostasis in embryonic and adult skin via distinct mechanisms. Mol Cell Biol.

[B6] Okushima Y, Mitina I, Quach HL, Theologis A (2005). AUXIN RESPONSE FACTOR 2 (ARF2): a pleiotropic developmental regulator. Plant J.

[B7] Kang HG, Fang Y, Singh KB (1999). A glucocorticoid-inducible transcription system causes severe growth defects in Arabidopsis and induces defense-related genes. Plant J.

[B8] Vreugdenhil D, Claassens MM, Verhees J, van der Krol AR, van der Plas LH (2006). Ethanol-inducible gene expression: non-transformed plants also respond to ethanol. Trends Plant Sci.

[B9] Tachibana C, Yoo JY, Tagne JB, Kacherovsky N, Lee TI, Young ET (2005). Combined Global Localization Analysis and Transcriptome Data Identify Genes That Are Directly Coregulated by Adr1 and Cat8. Mol Cell Biol.

[B10] Beyer A, Workman C, Hollunder J, Radke D, Möller U, Wilhelm T, Ideker T (2006). Integrated assessment and prediction of transcription factor binding. PLoS Comput Biol.

[B11] Kummerfeld SK, Teichmann SA (2006). DBD: a transcription factor prediction database. Nucleic Acids Res.

[B12] Vogel JT, Zarka DG, Van Buskirk HA, Fowler SG, Thomashow MF (2005). Roles of the CBF2 and ZAT12 transcription factors in configuring the low temperature transcriptome of Arabidopsis. Plant J.

[B13] Lemmens K, Dhollander T, De Bie T, Monsieurs P, Engelen K, Smets B, Winderickx J, De Moor B, Marchal K (2006). Inferring transcriptional modules from ChIP-chip, motif and microarray data. Genome Biol.

[B14] Shi Y, Mitchell T, Bar-Joseph Z (2007). Inferring pairwise regulatory relationships from multiple time series datasets. Bioinformatics.

[B15] Jen CH, Manfield IW, Michalopoulos I, Pinney JW, Willats WG, Gilmartin PM, Westhead DR (2006). The Arabidopsis co-expression tool (ACT): a WWW-based tool and database for microarray-based gene expression analysis. Plant J.

[B16] Steinhauser D, Usadel B, Luedemann A, Thimm O, Kopka J (2004). CSB.DB: a comprehensive systems-biology database. Bioinformatics.

[B17] Toufighi K, Brady SM, Austin R, Ly E, Provart NJ (2005). The Botany Array Resource: e-Northerns, Expression Angling, and promoter analyses. Plant J.

[B18] Obayashi T, Kinoshita K, Nakai K, Shibaoka M, Hayashi S, Saeki M, Shibata D, Saito K, Ohta H (2007). ATTED-II: a database of co-expressed genes and cis elements for identifying co-regulated gene groups in Arabidopsis. Nucleic Acids Res.

[B19] Persson S, Wei H, Milne J, Page G, Somerville C (2005). Identification of genes required for cellulose synthesis by regression analysis of public microarray data sets. Proc Natl Acad Sci USA.

[B20] Gachon CM, Langlois-Meurinne M, Henry Y, Saindrenan P (2005). Transcriptional co-regulation of secondary metabolism enzymes in Arabidopsis: functional and evolutionary implications. Plant Mol Biol.

[B21] Kurata T, Okada K, Wada T (2005). Intercellular movement of transcription factors. Curr Opin Plant Biol.

[B22] Liu H, Colavitti R, Rovira II, Finkel T (2005). Redox-dependent transcriptional regulation. Circ Res.

[B23] Yu H, Luscombe NM, Qian J, Gerstein M (2003). Genomic analysis of gene expression relationships in transcriptional regulatory networks. Trends Genet.

[B24] Reiss D, Baliga N, Bonneau R (2006). Integrated biclustering of heterogeneous genome-wide datasets for the inference of global regulatory networks. BMC Bioinformatics.

[B25] Ji L, Tan KL (2005). Identifying time-lagged gene clusters using gene expression data. Bioinformatics.

[B26] Balasubramaniyan R, Hüllermeier E, Weskamp N, Kämper J (2005). Clustering of gene expression data using a local shape-based similarity measure. Bioinformatics.

[B27] Costa IG, Schönhuth A, Schliep A (2005). The Graphical Query Language: a tool for analysis of gene expression time-courses. Bioinformatics.

[B28] Heard NA, Holmes CC, Stephens DA, Hand DJ, Dimopoulos G (2005). Bayesian coclustering of Anopheles gene expression time series: study of immune defense response to multiple experimental challenges. Proc Natl Acad Sci USA.

[B29] Gasch AP, Eisen MB (2002). Exploring the conditional coregulation of yeast gene expression through fuzzy k-means clustering. Genome Biol.

[B30] Bläsing OE, Gibon Y, Gunther M, Höhne M, Morcuende R, Osuna D, Thimm O, Usadel B, Scheible WR, Stitt M (2005). Sugars and circadian regulation make major contributions to the global regulation of diurnal gene expression in Arabidopsis. Plant Cell.

[B31] Hastie T, Tibshirani R, Eisen MB, Alizadeh A, Levy R, Staudt L, Chan WC, Botstein D, Brown P (2000). 'Gene shaving' as a method for identifying distinct sets of genes with similar expression patterns. Genome Biol.

[B32] Kilian J, Whitehead D, Horak J, Wanke D, Weinl S, Batistic O, D'Angelo C, Bornberg-Bauer E, Kudla J, Harter K (2007). The AtGenExpress global stress expression data set: protocols, evaluation and model data analysis of UV-B light, drought and cold stress responses. Plant J.

[B33] Seki M, Narusaka M, Ishida J, Nanjo T, Fujita M, Oono Y, Kamiya A, Nakajima M, Enju A, Sakurai T, Satou M, Akiyama K, Taji T, Yamaguchi-Shinozaki K, Carninci P, Kawai J, Hayashizaki Y, Shinozaki K (2002). Monitoring the expression profiles of 7000 Arabidopsis genes under drought, cold and high-salinity stresses using a full-length cDNA microarray. Plant J.

[B34] Fowler S, Thomashow MF (2002). Arabidopsis transcriptome profiling indicates that multiple regulatory pathways are activated during cold acclimation in addition to the CBF cold response pathway. Plant Cell.

[B35] Bar-Joseph Z, Gerber G, Gifford D, Jaakkola T, Simon I (2003). Continuous representations of time-series gene expression dat. J Comput Biol.

[B36] Abe H, Yamaguchi-Shinozaki K, Urao T, Iwasaki T, Hosokawa D, Shinozaki K (1997). Role of arabidopsis MYC and MYB homologs in drought- and abscisic acid-regulated gene expression. Plant Cell.

[B37] Chattopadhyay S, Ang LH, Puente P, Deng XW, Wei N (1998). Arabidopsis bZIP protein HY5 directly interacts with light-responsive promoters in mediating light control of gene expression. Plant Cell.

[B38] Maruyama K, Sakuma Y, Kasuga M, Ito Y, Seki M, Goda H, Shimada Y, Yoshida S, Shinozaki K, Yamaguchi-Shinozaki K (2004). Identification of cold-inducible downstream genes of the Arabidopsis DREB1A/CBF3 transcriptional factor using two microarray systems. Plant J.

[B39] Suzuki N, Rizhsky L, Liang H, Shuman J, Shulaev V, Mittler R (2005). Enhanced tolerance to environmental stress in transgenic plants expressing the transcriptional coactivator multiprotein bridging factor 1c. Plant Physiol.

[B40] Abe H, Urao T, Ito T, Seki M, Shinozaki K, Yamaguchi-Shinozaki K (2003). Arabidopsis AtMYC2 (bHLH) and AtMYB2 (MYB) function as transcriptional activators in abscisic acid signaling. Plant Cell.

[B41] Busch W, Wunderlich M, Schöff F (2005). Identification of novel heat shock factor-dependent genes and biochemical pathways in Arabidopsis thaliana. Plant J.

[B42] Gilmour SJ, Fowler SG, Thomashow MF (2004). Arabidopsis transcriptional activators CBF1, CBF2, and CBF3 have matching functional activities. Plant Mol Biol.

[B43] Sakuma Y, Maruyama K, Osakabe Y, Qin F, Seki M, Shinozaki K, Yamaguchi-Shinozaki K (2006). Functional analysis of an Arabidopsis transcription factor, DREB2A, involved in drought-responsive gene expression. Plant Cell.

[B44] Fujita Y, Fujita M, Satoh R, Maruyama K, Parvez MM, Seki M, Hiratsu K, Ohme-Takagi M, Shinozaki K, Yamaguchi-Shinozaki K (2005). AREB1 is a transcription activator of novel ABRE-dependent ABA signaling that enhances drought stress tolerance in Arabidopsis. Plant Cell.

[B45] van Buskirk H, Thomashow M (2006). Arabidopsis transcription factors regulating cold acclimation. Physiol Plantarum.

[B46] Oravecz A, Baumann A, Máté Z, Brzezinska A, Molinier J, Oakeley EJ, Adám E, Schäfer E, Nagy F, Ulm R (2006). CONSTITUTIVELY PHOTOMORPHOGENIC1 is required for the UV-B response in Arabidopsis. Plant Cell.

[B47] Tohge T, Nishiyama Y, Hirai MY, Yano M, Nakajima J, Awazuhara M, Inoue E, Takahashi H, Goodenowe DB, Kitayama M, Noji M, Yamazaki M, Saito K (2005). Functional genomics by integrated analysis of metabolome and transcriptome of Arabidopsis plants over-expressing a MYB transcription factor. Plant J.

[B48] Gibbons FD, Roth FP (2002). Judging the quality of gene expression-based clustering methods using gene annotation. Genome Res.

[B49] Steuer R, Humburg P, Selbig J (2006). Validation and functional annotation of expression-based clusters based on gene ontology. BMC Bioinformatics.

[B50] van Helden J (2003). Regulatory sequence analysis tools. Nucleic Acids Res.

[B51] Thijs G, Lescot M, Marchal K, Rombauts S, Moor BD, Rouzé P, Moreau Y (2001). A higher-order background model improves the detection of promoter regulatory elements by Gibbs sampling. Bioinformatics.

[B52] Bailey TL, Williams N, Misleh C, Li WW (2006). MEME: discovering and analyzing DNA and protein sequence motifs. Nucleic Acids Res.

[B53] Tompa M, Li N, Bailey T, Church G, De Moor B, Eskin E, Favorov A, Frith M, Fu Y, Kent W, Makeev V, Mironov A, Noble W, Pavesi G, Pesole G, Régnier M, Simonis N, Sinha S, Thijs G, van Helden J, Vandenbogaert M, Weng Z, Workman C, Ye C, Zhu Z (2005). Assessing computational tools for the discovery of transcription factor binding sites. Nat Biotechnol.

[B54] Pavesi G, Mereghetti P, Mauri G, Pesole G (2004). Weeder Web: discovery of transcription factor binding sites in a set of sequences from co-regulated genes. Nucleic Acids Res.

[B55] Davuluri R, Sun H, Palaniswamy S, Matthews N, Molina C, Kurtz M, Grotewold E (2003). AGRIS: Arabidopsis gene regulatory information server, an information resource of Arabidopsis cis-regulatory elements and transcription factors. BMC Bioinformatics.

[B56] Thimm O, Bläsing O, Gibon Y, Nagel A, Meyer S, Kruger P, Selbig J, Muller LA, Rhee SY, Stitt M (2004). MAPMAN: a user-driven tool to display genomics data sets onto diagrams of metabolic pathways and other biological processes. Plant J.

[B57] Wold S, Sjöstrom M, Eriksson L (2001). PLS-regression a basic tool of chemometrics. Chemom Intell Lab Syst.

[B58] Höskuldsson A (1988). PLS regression methods. J Chemom.

[B59] TAIR (2000). The Arabidopsis Information Resource. http://www.arabidopsis.org.

[B60] Irizarry R, Bolstad B, Collin F, Cope L, Hobbs B, Speed T (2003). Summaries of Affymetrix GeneChip probe level data. Nucleic Acids Res.

[B61] Gentleman R, Carey V, Bates D, Bolstad B, Dettling M, Dudoit S, Ellis B, Gautier L, Ge Y, Gentry J, Hornik K, Hothorn T, Huber W, Iacus S, Irizarry R, Li FLC, Maechler M, Rossini A, Sawitzki G, Smith C, Smyth G, Tierney L, Yang J, Zhang J (2004). Bioconductor: Open software development for computational biology and bioinformatics. Genome Biol.

[B62] R Development Core Team (2004). R: A language and environment for statistical computing.

[B63] Benjamini Y, Hochberg Y (1995). Controlling the false discovery rate: a practical and powerful approach to multiple testing. J R Stat Soc.

[B64] Hannah MA, Heyer AG, Hincha DK (2005). A Global Survey of Gene Regulation during Cold Acclimation in Arabidopsis thaliana. PLoS Genet.

[B65] Usadel B, Nagel A, Thimm O, Redestig H, Blaesing OE, Palacios-Rojas N, Selbig J, Hannemann J, Piques MC, Steinhauser D, Scheible WR, Gibon Y, Morcuende R, Weicht D, Meyer S, Stitt M (2005). Extension of the visualization tool MapMan to allow statistical analysis of arrays, display of corresponding genes, and comparison with known responses. Plant Physiol.

[B66] The Gene Ontology Consortium (2000). Gene Ontology: tool for the unification of biology. Nat Genet.

